# Cholecystokinin receptor-1 mediates the inhibitory effects of exogenous cholecystokinin octapeptide on cellular morphine dependence

**DOI:** 10.1186/1471-2202-13-63

**Published:** 2012-06-08

**Authors:** Di Wen, Chun-ling Ma, Ya-jing Zhang, Yan-xin Meng, Zhi-yu Ni, Shu-jin Li, Bin Cong

**Affiliations:** 1 Department of Forensic Medicine, Hebei Medical University, Hebei Key Laboratory of Forensic Medicine, Shijiazhuang, 050017, PR China; 2 The First Hospital of Shijiazhuang, Shijiazhuang, 050011, PR China

**Keywords:** Cholecystokinin octapeptide, CCK1 receptor, CCK2 receptor, Morphine, Cellular dependence, cAMP overshoot

## Abstract

**Background:**

Cholecystokinin octapeptide (CCK-8), the most potent endogenous anti-opioid peptide, has been shown to regulate the processes of morphine dependence. In our previous study, we found that exogenous CCK-8 attenuated naloxone induced withdrawal symptoms. To investigate the precise effect of exogenous CCK-8 and the role of cholecystokinin (CCK) 1 and/or 2 receptors in morphine dependence, a SH-SY5Y cell model was employed, in which the μ-opioid receptor, CCK1/2 receptors, and endogenous CCK are co-expressed.

**Results:**

Forty-eight hours after treating SH-SY5Y cells with morphine (10 μM), naloxone (10 μM) induced a cAMP overshoot, indicating that cellular morphine dependence had been induced. The CCK receptor and endogenous CCK were up-regulated after chronic morphine exposure. The CCK2 receptor antagonist (LY-288,513) at 1–10 μM inhibited the naloxone-precipitated cAMP overshoot, but the CCK1 receptor antagonist (L-364,718) did not. Interestingly, CCK-8 (0.1-1 μM), a strong CCK receptor agonist, dose-dependently inhibited the naloxone-precipitated cAMP overshoot in SH-SY5Y cells when co-pretreated with morphine. The L-364,718 significantly blocked the inhibitory effect of exogenous CCK-8 on the cAMP overshoot at 1–10 μM, while the LY-288,513 did not. Therefore, the CCK2 receptor appears to be necessary for low concentrations of endogenous CCK to potentiate morphine dependence in SH-SY5Y cells. An additional inhibitory effect of CCK-8 at higher concentrations appears to involve the CCK1 receptor.

**Conclusions:**

This study reveals the difference between exogenous CCK-8 and endogenous CCK effects on the development of morphine dependence, and provides the first evidence for the participation of the CCK1 receptor in the inhibitory effects of exogenous CCK-8 on morphine dependence.

## Background

Opioids are not only potent analgesics but are also drugs of abuse, which greatly limits their clinical use. Chronic use of opioids results in the development of tolerance and dependence. Recent studies suggest that non-opioid systems could be important targets for the treatment of opioid dependence [1,2]. Antagonism of opioid effects by endogenous anti-opioid peptides is common [3]. Cholecystokinin (CCK) was initially identified as a gastrointestinal hormone, and was subsequently found in the central and peripheral nervous systems. It occurs as various sized peptides, including 4, 8, 33, 39 and 58 amino acid forms [4]. Cholecystokinin octapeptide (CCK-8) is the most potent endogenous anti-opioid peptide [5]. For example, morphine treatments enhance the overflow of CCK, whereas CCK-8 and its analogues attenuate the analgesic effects of morphine [6-8]. Furthermore, studies have demonstrated that the CCK system modulates a variety of physiological processes, and that CCK-8 interacts with the GABAergic and dopaminergic systems. Thus, CCK-8 plays a significant role in a wide range of central actions including memory and drug reward [9-12]. Overlap in the distributions of CCK and opioid receptors may be observed in some anatomical regions that are involved in opioid antinociception and dependence [13]. These findings indicate that the CCK system is related to the modulation of morphine tolerance and dependence.

The administration of CCK receptor antagonists can prevent or reverse tolerance to systemic exogenous opioids or electroacupuncture-induced analgesia, as well as suppress morphine withdrawal syndromes [14-16]. Interestingly, CCK receptor activation can reverse morphine tolerance [17]. We previously found that chronic pretreatment with exogenous CCK-8 significantly inhibited naloxone-precipitated withdrawal symptoms, which is the same effect as CCK-receptor antagonists [18,19]. These phenomena suggest that the effects of exogenous CCK-8 are distinct from the role of endogenous CCK. With regard to dosage, CCK-8 was able to prevent morphine dependence at high, but not low, concentrations [20]. However, no studies have reported a dose–response curve of CCK-8 on morphine dependence. On the basis of the pharmacological properties and specificities for ligand binding, CCK receptors have been identified for the CCK1 and CCK2 receptor subtypes [21]. The expression pattern of these CCK receptors in mammals appears to be tissue-specific [22]. Several studies have revealed that two different CCK receptors (CCK1 and CCK2) have opposing influences on behavioural and hormonal actions [23,24]. Thus, CCK receptor subtypes that mediate the inhibitory effects of exogenous CCK-8 on morphine dependence remain to be determined.

The biological basis of tolerance and dependence induced by chronic exposure to opioids is thought to be due to molecular, cellular, and neural network adaptations. On the cellular level, opioid dependence is characterised by a significant elevation of adenylyl cyclase (AC) activity after drug withdrawal, which is a regulatory phenomenon termed "AC supersensitivity" or "cAMP overshoot" [25-27]. A cAMP overshoot represents an opioid-dependent state *in vitro* and has been utilised in the study of the effects of morphine dependence [28]. This present study evaluates the effects of CCK-8 on the AMP overshoot based on a cellular model and aims to clarify the exact effects of exogenous CCK-8. In addition, the subtypes of CCK receptor that mediate the function of CCK-8 on morphine dependence are also investigated.

## Results

### *In vitro* model of morphine dependence

**Figure 1:**
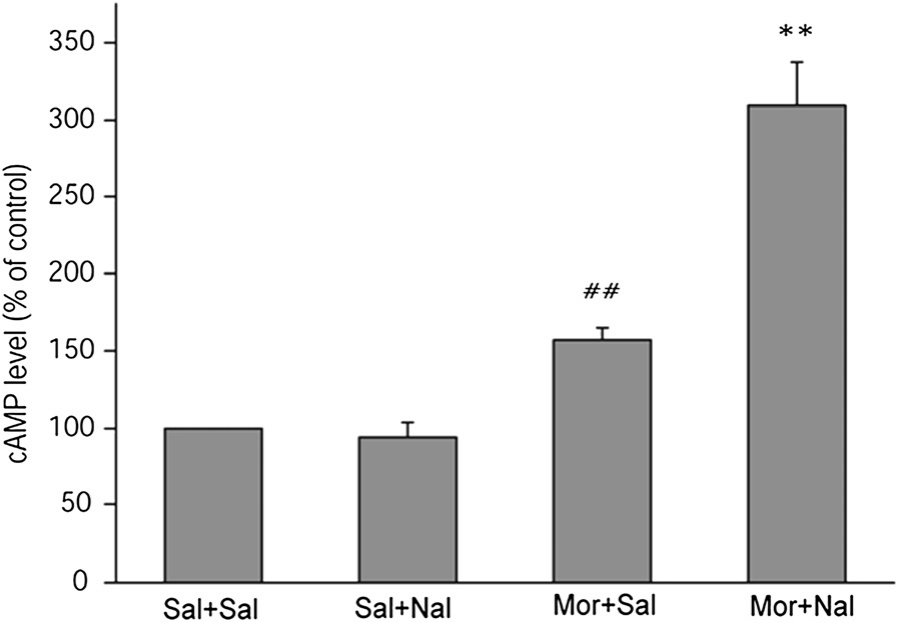
**The SH-SY5Y cells were pretreated with morphine (10 μM) exposure for 48 h, precipitated with naloxone (10 μM) for 15 min, and then the levels of cAMP were measured.** The data are from the analysis of the ratio of the levels of cAMP relative to the control (Sal+Sal), and are presented as the mean ± S.D. of three separate experiments. ##*P* < 0.01, compared with the Sal + Sal group; **P < 0.01, compared with the Mor + Sal group.

The SH-SY5Y cell line was used to examine the effects of CCK-8 on cellular morphine dependence. It is well established that up-regulation of the adenylate cyclase effector system represents an *in vitro* opioid-dependent state. Thus, withdrawal of opioids following chronic treatment leads to a cAMP overshoot, which is also referred to ‘cAMP super-sensitisation’, and is an *in vitro* model of the abstinence state. In the present study, the level of cAMP increased 1.57 (±0.08)-folds after morphine treatment (10 μM; 48 h), and was elevated to 3.09 (±0.289)-folds after naloxone treatment, compared to the non-treated cells (10 μM; 15 min). The analysis of the data obtained from the cAMP tests with two-way ANOVA revealed a significant morphine and naloxone interaction (F_1, 8_ = 53.718, *P* < 0.001) on the cAMP level, which indicated that the cAMP overshoot was significantly induced by chronic morphine exposure following naloxone precipitation (Figure 1).

### The effect of chronic morphine exposure on the endogenous CCK system

**Figure 2:**
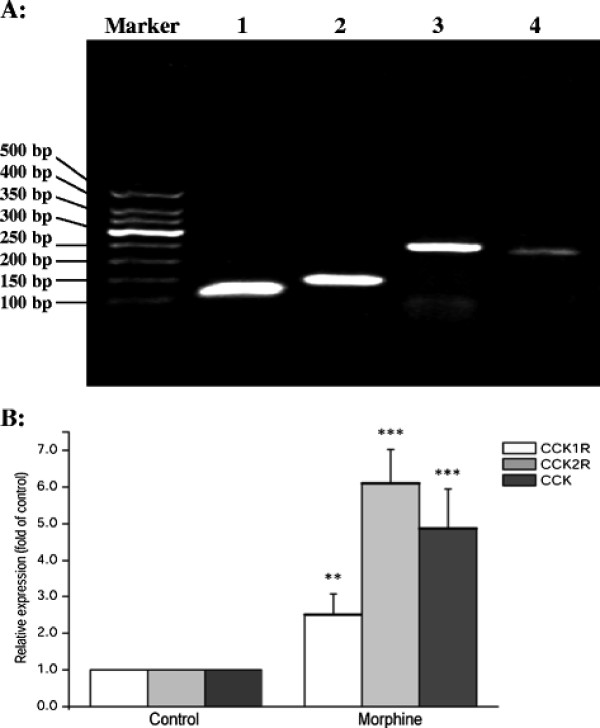
**A: MOR (μ-opioid receptor), CCK1R (CCK1 receptor), CCK2R (CCK2 receptor) and CCK mRNA are co-expressed in SH-SY5Y cells.** The PCR products were separated by 2% agarose gel electrophoresisi and imaged. Lane 1: MOR; Lane 2: CCK1R; Lane 3: CCK2R; Lane 4: CCK. **B**: The effects of chronic morphine exposure (10 μM) for 48 h on CCK1R, CCK2R and endogenous CCK expression. The data are obtained from the analysis of the fold of relative expression level compared to the saline control group, and expressed as the mean ± S.D. of three independent experiments. **P<0.01, ***P<0.001 compared with the control by *t*-test.

The expression of the opioid and CCK system in SH-SY5Y cells was initially confirmed. Our results revealed that the μ-opioid, CCK1 and CCK2 receptors were co-expressed in SH-SY5Y cells, and that SH-SY5Y cells contained endogenous CCK (Figure 2A). The effect of chronic morphine exposure on the expression of the endogenous CCK system in SH-SY5Y cells was measured. The expression of the CCK1/CCK2 receptor and endogenous CCK mRNA were up-regulated 2.51(± 0.56)-, 6.10(± 0.91)- and 4.87(± 1.08)-fold (*P* = 0.004, *P* < 0.001 and *P* < 0.001, respectively) after 10 μM morphine treatment for 48 h (Figure 2B).

### The effect of CCK receptor antagonists on naloxone-precipitated cAMP overshoot in SH-SY5Y cells after chronic morphine exposure

**Figure 3:**
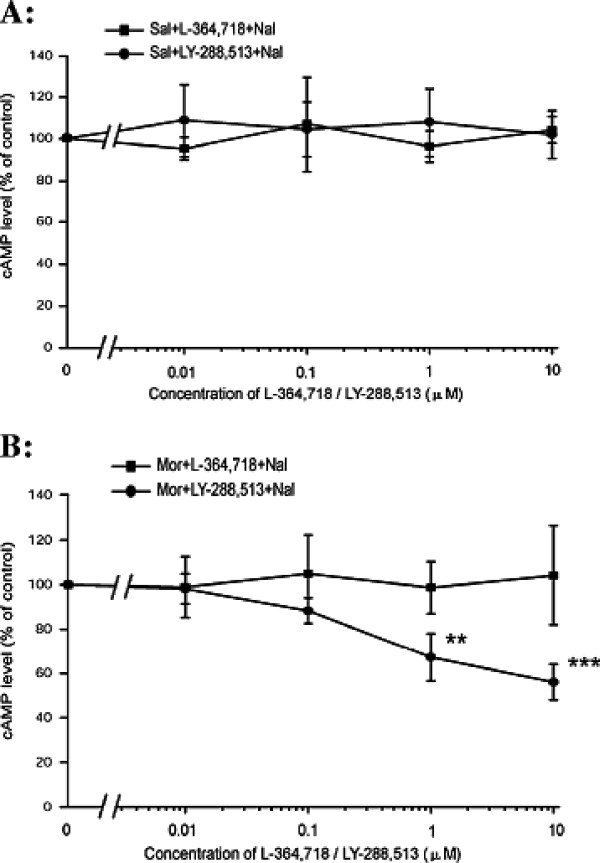
**The effect of L-364,718 or LY-288,513 on naloxone precipitated cAMP overshoot in saline (A) or morphine (B) pretreated SH-SY5Y cells.** The SH-SY5Y cells were first co-pretreated with L-364,718 or LY-288,513 (0-10 μM) and saline for 48 h to evaluate the effect of CCK receptor antagonists on the baseline cAMP level, and then incubated with L-364,718 or LY-288,513 plus morphine (10 μM) to investigate the influence of CCK receptor antagonists on the naloxone (10 μM, 15 min) precipitated cAMP overshoot. The control groups were considered as no CCK receptor antagonist-treated cells (0 μM L-364,718/LY-288,513) in each independent experiment. The results of the cAMP levels are represented as the percentage of cAMP content relative to the control group. The data are presented as the mean ± S.D. of three separate experiments, performed in duplicate. **P < 0.01, ***P < 0.01, compared with the control by one-way ANOVA followed by Dunnett’s *t*-test.

CCK-8 is the most potent endogenous anti-opioid peptide and endogenous CCK expression is up-regulated after chronic morphine treatment. Therefore, L-364,718, a CCK1 receptor antagonist, and LY-288,513, a CCK2 receptor antagonist (0.01-10 μM), were utilised to examine endogenous CCK regulation of the development of morphine dependence in SH-SY5Y cells. Baseline experiments were first conducted in the absence of the opioid. Pretreatment with L-364,718 (F_4, 10_ = 0.593, *P* = 0.676) or LY-288,513 (F_4, 10_ = 0.246, *P* = 0.905) alone did not affect cellular basal cAMP levels (Figure 3A). Co-pretreatment of LY-288,513 (1 and 10 μM) with morphine (10 μM) for 48 h significantly inhibited cAMP overshoot following naloxone (10 μM, 15 min) precipitation (F_4, 10_ = 22.185, *P* < 0.001, Figure 3B), however, L-364,718 exhibited no effect (F_4,10_ = 0.119, *P* = 0.972, Figure 3B). Consistent with a previous study *in vivo*, these findings suggest that endogenous CCKs have a notable effect on morphine dependence *in vitro via* the CCK2 receptor.

### The effect of exogenous CCK-8 on naloxone-precipitated cAMP overshoot in SH-SY5Y cells after chronic morphine exposure

**Figure 4:**
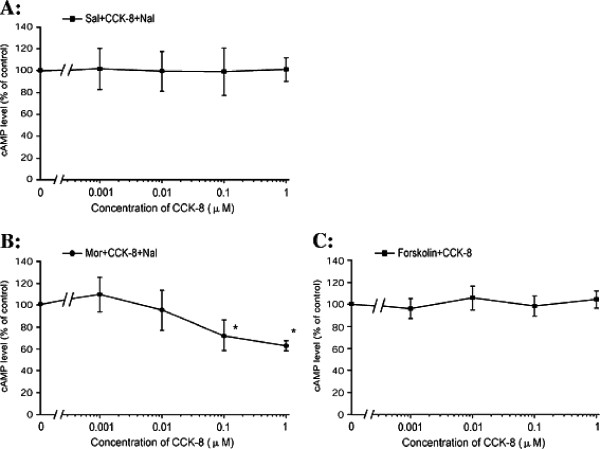
**The effects of CCK-8 on saline (A) or morphine (B) pretreated, naloxone precipitated cAMP overshoot and, on forskolin stimulated cAMP accumulation (C) in SH-SY5Y cells.** The SH-SY5Y cells were co-pretreated with CCK-8 (0.001-10 μM) and saline or 10 μM morphine for 48 h to observe the effect of exogenous CCK-8 on the baseline cAMP levels and morphine withdrawal-induced cAMP overshoot, respectively. The cells were then co-pretreated with CCK-8 (0.001-10 μM) and forskolin (10 μM) to rule out non-specific inhibition of CCK-8 on the cAMP overshoot. The control groups were as the cells that received no CCK-8 (0 μM CCK-8) treatment in each independent experiment. The results of the cAMP levels are represented as the percentage of cAMP content relative to the control group. The data are presented as the mean ± S.D. of three separate experiments, performed in duplicate. *P < 0.05, compared with the control by one-way ANOVA followed by Dunnett’s *t*-test.

Exogenous CCK-8 (0.1-1 μM), a CCK receptor agonist, inhibited the naloxone (10 μM) precipitated cAMP overshoot when co-pretreated with morphine (10 μM; 48 h) (F_4, 10_ = 8.914, *P* = 0.002, Figure 4B) in a dose-dependent manner, indicating that exogenous CCK-8 has an identical regulatory effect on cellular morphine dependence as the antagonist. A significant inhibitory effect was observed at a concentration of 0.1 μM (*P* = 0.028) and 1 μM (*P* = 0.011), but not at 0.001 μM or 0.1 μM (*P* = 0.722, *P* = 0.967), compared with the morphine pre-treated control cells. Baseline experiments were conducted in the absence of the opioid. Treatment with CCK-8 at concentrations from 0.001 to 1 μM for 48 h exhibited no effect on basal cAMP levels (F_4, 10_ = 0.132, *P* = 0.967, Figure 4A). Moreover, exogenous CCK-8 did not alter the forskolin stimulated cAMP levels (F_4, 10_ = 2.685, *P* = 0.094, Figure 4C), indicating that a non-specific inhibitory mechanism on cAMP shoot induced by CCK-8 could be ruled out.

### Blockade of the CCK1 receptor attenuates the effect of exogenous CCK-8 on naloxone-precipitated cAMP overshoot after chronic morphine exposure

**Figure 5:**
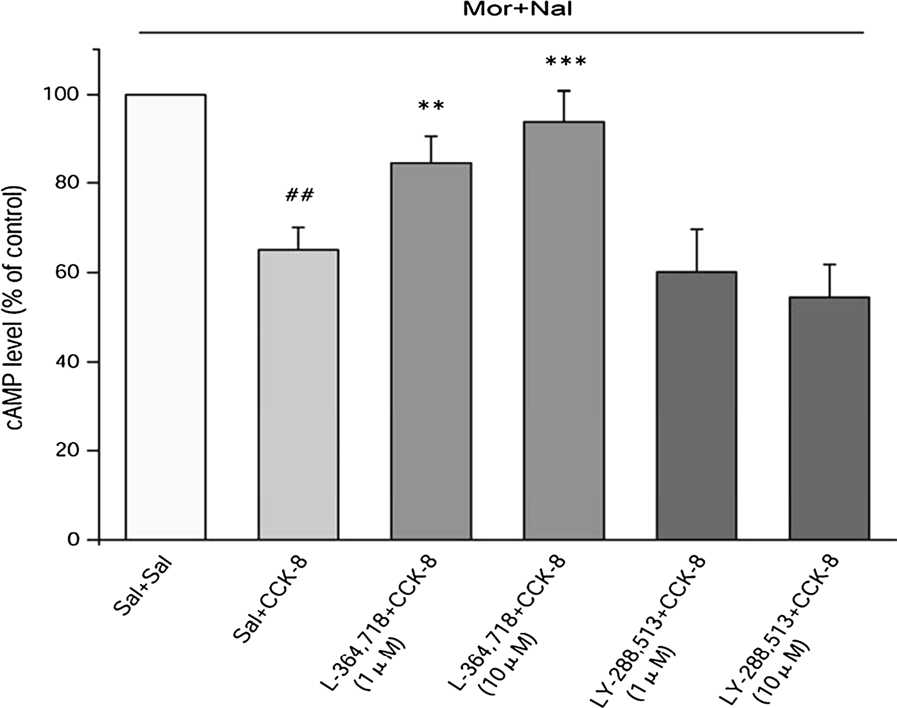
**The CCK1 receptor antagonist blocked the inhibitory effects of exogenous CCK-8 on naloxone precipitated cAMP overshoot after chronic morphine exposure.** The SH-SY5Y cells were co-pretreated with L-364,718(1 and 10 μM) or LY-288,513(1 and 10 μM) and CCK-8 (1 μM) plus morphine (10 μM) for 48 h. After the removal of the drugs, naloxone (10 μM, 15 min) was added for the induction of the cAMP overshoot. The control group was the no CCK-8 and CCK receptor antagonist treated cells (Sal + Sal). The results of the cAMP level are represented as the percentage of cAMP content relative to the control group. The data are the mean ± S.D. of three separate experiments, performed in duplicate. ##P < 0.01, compared with the Sal + Sal group by *t*-test; **P < 0.01, ***P < 0.001 as compared with the Sal + CCK-8 group by one-way ANOVA followed by Dunnett’s *t*-test.

As described above, CCK-8 (0.1-1 μM) inhibited naloxone precipitated cAMP overshoot when co-pretreated with morphine in a dose-dependent manner. We then blocked CCK1 and CCK2 receptors using L-364,718 and LY-288,513, respectively, to determine the role of CCK receptor subtypes in exogenous CCK-8 regulating morphine dependence. The cells were pre-treated with L-364,718 (1 and 10 μM) or LY-288,513 (1 and 10 μM), 15 min before co-treatment of morphine (10 μM) and CCK-8 (1 μM). Next, the cAMP overshoot was subsequently induced by adding 10 μM naloxone. The inhibitory effect of CCK-8 on the naloxone precipitated cAMP overshoot was significantly reduced at both 1 μM (*P* = 0.003) and 10 μM (*P* < 0.001) L-364,718 pre-treated cells (Figure 5). However, no change was observed in 1 μM (*P* = 0.373) and 10 μM (*P* = 0.069) LY-288,513 pre-treated cells, indicating that the CCK1 receptor mediated the effect of exogenous CCK-8 on cellular morphine dependence. Due to the inhibitory function of LY-288,513 and CCK-8 on the cAMP overshoot (Figure 3 and Figure 4), a cumulative effect of LY-288,513 and exogenous CCK-8 was not observed.

## Discussion

In this present study, we describe a distinct effect of exogenous CCK-8 on the development of morphine dependence *in vitro*. Moreover, we provide the first piece of evidence that a CCK1 receptor antagonist can reverse the inhibitory effects of exogenous CCK-8 on morphine dependence. We also find that endogenous CCK exerts a potential facilitative effect *via* the CCK2 receptor. This suggests opposing roles of CCK1 and CCK2 receptors in the development of morphine dependence.

First, a suitable cell model was selected for this study. The SH-SY5Y cell line is derived from a human neuroblastoma cell line, SK-N-SH, by three rounds of subcloning. The SH-SY5Y cells are dopamine beta hydroxylase active, acetylcholinergic, glutamatergic and adenosinergic, and express abundant and functional μ- and δ-opioid receptors. SH-SY5Y cells have been extensively used for studies of opioid receptor regulation and intracellular signaling. Moreover, co-expression of the opioid and CCK systems in SH-SY5Y cells was confirmed, and CCK is endogenously expressed in SH-SY5Y cells. This system is useful for the study of the potential regulatory effects of exogenous CCK-8 on morphine dependence.

The interaction between CCK and opioids was first reported by Itoh *et al.* They showed that pre-treatment with CCK suppressed anti-nociception induced by β-endorphin [29]. A subsequent *in vivo* microdialysis study found that the extracellular levels of CCK significantly increased after morphine administration, thus acting as a negative feedback modulator and a potent anti-opioid peptide [6,7,30]. Studies have confirmed that endogenous CCK potentiates, and the CCK antagonist attenuates the tolerance and dependence of opioids [31,32]. The presence of opioid receptors in CCK-containing neurons suggest a potential direct influence of opioids on CCK release [33]. However, earlier studies have failed to show an affinity of CCK for opioid receptors, indicating that CCK does not behave as a classical receptor antagonist *via* binding to opioid receptors [34]. Han *et al.* found that the binding of CCK-8 to the CCK receptor reduces the binding affinity of μ-opioid receptor ligands, implying that receptor–receptor interaction between CCK and opioid systems may occur in an indirect manner [35]. The molecular cloning of CCK receptor subtypes, one from the pancreas (type-1) and another from human brain (type-2), has confirmed the pharmacological classification of CCK receptors. The CCK2 receptor is predominantly localised in the CNS, and mainly mediates anxiety, panic attacks, pain and drug dependence [36-38]. The CCK1 receptor is present in discrete regions of the brain and has a low affinity for central CCK [39], and its function is poorly understood with only a few reports investigating the central role in food intake regulation [40]. The use of highly selective receptor antagonists and antisense approaches has shown, at least in the rodent, that CCK2 receptors mediate the anti-opioid function of CCK [41-43]. The present study shows that co-pretreatment with LY-288,513 and morphine significantly inhibited the naloxone-precipitated cAMP overshoot in SH-SY5Y cells, but that co-pretreatment with L-364,718 displayed no effect. We verified that endogenous CCK played an anti-opioid role and potentiated the development of morphine dependence *via* the CCK2 receptor.

Together with our previous results, we found that exogenous CCK-8 pretreatment significantly inhibited morphine dependence *in vitro* and *in vivo*[44]. These results show that the treatments of the CCK receptor agonist and antagonist demonstrated the same effect on morphine dependence. Moreover, CCK-8 treatment did not affect basal or forskolin-stimulated cAMP levels, suggesting that the effect of exogenous CCK-8 was not simply a direct action on cAMP. Several studies have reported that small doses of CCK inhibit the anti-nociceptive action of opioids, whereas large doses of CCK induce analgesia [45]. We previously revealed that CCK-8 suppressed the binding affinity of the μ-opioid receptor in SH-SY5Y cells at concentrations of 1 nM, while it increased the expression of the endogenous opioid peptide from 0.1 to 1 μM [46]. The dose–response curve of CCK-8 was inversely U-shaped, and CCK-8 displayed a dose-dependent, biphasic effect [47]. We found that only high concentrations of CCK-8 were able to attenuate the cAMP overshoot in a dose-dependent manner. Thus, observations of exogenous CCK-8 may represent pharmacological effects, rather than physiological effects of endogenous CCK.

A previous study indicated that the CCK1 receptor was ineffective in the development of morphine dependence [42]. Nevertheless, we found that a high dose of exogenous CCK-8 markedly attenuated the naloxone precipitated cAMP overshoot *via* the CCK1 receptor. We concluded that the CCK1 and CCK2 receptors play unique and distinct roles in physiology and pathophysiology. Moreover, data showing that CCK1 receptors mediate mnemonic effects, and that CCK2 receptors mediate amnestic effects, have been reported [48]. Furthermore, CCK evokes [Ca^2+^_i_ signaling by the influx of extracellular calcium, likely through L-type calcium channels, and an antagonist for the CCK1 receptor blocked [Ca^2+^_i_ response to CCK-8 [49,50]. CCK produces direct neuronal depolarisation *via* CCK1 receptors and inhibits GABAergic synaptic transmission [51], while CCK2 receptor activation augments long-term potentiation in hippocampal slices [52]. CCK-8, the predominant central form of CCK, has a high affinity for the CCK2 receptor, but a low affinity for the CCK1 receptor. The CCK1 receptor is activated only in the presence of high CCK-8 levels, and exerts a different effect from the CCK2 receptor on morphine dependence. Due to the inhibitory function of LY-288,513 and CCK-8 on cAMP overshoot, the cumulative effect of LY-288,513 and exogenous CCK-8 on cAMP overshoot was not observed. The role of CCK2 receptor in the process of exogenous CCK-8 regulation on morphine dependence can not be ruled out.

## Conclusions

In conclusion, we identified a difference between the role of the CCK1 and CCK2 receptor on the development of morphine dependence and an inhibitory effect of high-doses of exogenous CCK-8 on cellular morphine dependence. In addition, this study provides the first evidence for the participation of the CCK1 receptor in the mechanism by which exogenous CCK-8 inhibits morphine dependence.

## Materials and methods

### Materials

Morphine hydrochloride was purchased from Shenyang First Pharmaceutical Factory (Liaoning, China). CCK-8, IBMX, naloxone and forskolin were purchased from Sigma (Sigma, St. Louis, MO, USA). Retinoic acid (RA) was purchased from Alfa Aesar (Alfa Aesar, Heysham, UK). The CCK1 receptor antagonist, L-364,718, and CCK2 receptor antagonist, LY-288,513, were purchased from Tocris Bioscience (Tocris Cookson, Northpoint, UK). DMEM/F12 medium was purchased from Invitrogen Corporation (Gibco™, Grand Island, NY, USA). Fetal bovine serum was purchased from PAA Laboratories (PAA, Strasse, Pasching, Austria). LANCE™ cAMP kits were purchased from PerkinElmer (PerkinElmer, MA, USA). CCK-8 was suspended in vehicle consisting of 1% ammonia saline solution at 1 mg/ml, and the CCK receptor antagonists were suspended in DMSO at 1 mg/ml.

### Cell culture

Human SH-SY5Y neuroblastoma cells were obtained from Shanghai Bioleaf Biotec (Shanghai, China). The cells were seeded at 1 × 10^6^ cell/25 cm^2^ in tissue culture flasks and grown in DMEM/F12 medium supplemented with 10% fetal bovine serum, 100 U/ml penicillin and 100 U/ml streptomycin for 24 hours. The cells were then morphologically differentiated into neuron-like cells by treatment with10 mM RA. Similar with previous studies [53], the RA-containing medium was replaced every 2 days. The cultured SH-SY5Y cells were then used for experiments 6 days after the initiation of differentiation. All of the cultures were maintained at 37 °C in a humidified atmosphere consisting of 95% air and 5% CO_2_.

### Real-time PCR analysis

The cells were harvested and the total RNA was extracted with TriZol Reagent (Invitrogen, Carlsbad, CA) according to the manufacturer’s instructions. The RNA concentrations were determined using a Nanodrop ND-1000 spectrophotometer (Nanodrop Technologies, Wilmington, DE, USA), and the complementary DNA (cDNA) was synthesised from the total RNA (500 ng) using the PrimeScript™ RT regent Kit (Takara Biotechnology, Dalian, China) following the instructions provided by the manufacturer. Subsequently, the cDNA was subjected to real-time PCR using the Power SYBR Green PCR Master Mix (Takara Biotechnology, Dalian, China). Each real-time PCR reaction consisted of 2 μl of diluted RT product, 10 μl SYBR Green PCR Master Mix and 250-nm specific primer pairs (Table 1) in a total volume of 20 μl. The reactions were performed on a 7500 real-time PCR System (Applied Biosystems) for 40 cycles (95 °C for 5 s, 60 °C for 35 s) after an initial 30 s incubation at 95 °C. The PCR products were separated by 2% agarose gel electrophoresis, illuminated with UV light and imaged to assess the amplification. The fold change in the mRNA levels of each gene was calculated using the ΔΔCt method, with the house keeping gene, β-actin, as an internal control.

**Table 1:** Primer sequences used for real-time PCR

**Gene**	**Sense**	**Anti-sense**
**MOR**	5’-ATCACGATCATGGCCCTCTACTCC-3’	5’-TGGTGGCAGTCTTCATCTTGGTGT-3’
**CCK1R**	5’-GACGCTTCGGTCATTAGA-3’	5’-AGGGAGGAGTGATGTTGC-3’
**CCK2R**	5’-CCCACTCCCTCCATTGCT-3’	5’-CTGCTCCATTCTTATTCCTCTT-3’
**CCK**	5’-AGCTGAGGGTATCGCAGAGA-3’	5’-TGGGTCCTCTAGGAGGGGTA-3’
**β-actin**	5’-GGGACCT GACTGACTACCTC-3’	5’-ACTCGTCATACTCCTGCTTG-3’

### cAMP accumulation assays

The differentiated SH-SY5Y cells that had been treated with morphine and/or CCK-8 for 48 h were assayed for cAMP accumulation using a LANCE™ cAMP kit (PerkinElmer). The cells were harvested with a Versene dissociation solution followed by washing with HBSS buffer. The cells were then resuspended at a concentration of 2 × 10^6^ cells/ml in stimulation buffer (HBSS 1×, containing 5 mM HEPES, 0.1% BSA, 0.05 mM IBMX). The Alexa pluor®647 labeled antibodies were added to the final cell suspension, and then naloxone was added to the cell suspension to precipitate the cAMP overshoot. After incubation at 37 °C for 15 min, the Detection Mix was added to the mixture. The sample was further incubated for 1 h, and was read on a TECAN instrument (Infinitie F200, Tecan, Grodig, Austria) to measure the LANCE signal. Simultaneous to the measurement of the cell-based cAMP level, the cAMP standard curve was assayed according to the manufacturer’s instructions. The LANCE signal obtained at 665 nM can be directly used to analyse the cAMP levels. The signal at 615 nM is useful to identify dispensing or quenching problems.

### Statistical analysis

The data are presented as the mean ± standard deviation (S.D.) All of the experiments were performed at least three times, each with a different culture. The statistical analyses (SPSS, v. 13.0, Chicago, USA) were performed with two-way ANOVA to evaluate the interaction between morphine and naloxone on the cAMP overshoot to estabish the cellular model of morphine dependence, and then with *t*-test and one-way ANOVA followed by Dunnett’s *t*-test for subsequent experiments. A *P*-value <0.05 was considered statistically significant.

## Authors’ contributions

All of the authors contributed to the writing of the manuscript and approved of the final version. DW performed the experiments and data analysis, participated in the design of the study and drafted the manuscript. CLM and BC conceived of and designed the study and supervised the work. YJZ and YXM contributed to the design of the study, and helped with performing the experiments and drafting the manuscript. ZYN and SJL provided intellectual input to the study and helped with the revision of the manuscript. All authors read and approved the final manuscript
